# Manifestation and sociodemographic microdata of Brazil’s Unified Health System Ombudsman

**DOI:** 10.1186/s13104-023-06639-x

**Published:** 2024-01-05

**Authors:** Raphael de Freitas Saldanha, Vinicius Kreischer, Raquel Gritz, Matheus Miloski, Carmen Lúcia Corrêa Bonifácio, Carlos Leonardo Souza Cardoso, Ariane Camilo Pinheiro Alves, Domício Aurélio de Sá, Garibaldi Dantas Gurgel Junior, Rafael da Silveira Moreira, Michelle Vieira Fernandez, Jefferson da Costa Lima, Marcel Pedroso

**Affiliations:** 1 Platform of Data Science applied to Health (PCDaS), ICICT, Oswaldo Cruz Foundation, Fiocruz, Rio de Janeiro, Brazil; 2 Data Extreme Lab, National Laboratory for Scientific Computing, DEXL Lab/LNCC, Petrópolis, Brazil; 3 National Laboratory for Scientific Computing, LNCC, Petrópolis, Brazil; 4 Federal University of Rio de Janeiro, UFRJ, Rio de Janeiro, Brazil; 5 Aggeu Magalhães Institute, IAM, Pernambuco, Brazil

**Keywords:** Ombudsman, Brazilian Unified Health System, Health informatics, Data science

## Abstract

**Objectives:**

This article presents the process of extraction and treatment of two datasets from the General Ombudsman of the Brazilian Unified Health System (OUVSUS). The resulting datasets allow the analysis of manifestation characteristics and sociodemographic profile of the citizens that performed these manifestations.

**Data description:**

The first dataset depicts the characteristics of the manifestations registered by the General Ombudsman. Each row represents an individual manifestation and contains information such as the registration date, classification, input channel, and subject, among others. The second dataset is constituted of sociodemographic information for each citizen that performed a manifestation, and characteristics such as sexual orientation, race, age, and geographic location of the citizen are presented, among others.

## Objective

This article presents the process of creation, treatment, and availability of two datasets from the General Ombudsman of the Brazilian Unified Health System (OUVSUS).

The General Ombudsman works permanently, through direct communication with citizens, to enable social participation, the dissemination of health information, and formal mediation between the needs of users and the managers of the Brazilian Unified Health System (SUS) [1].

Different channels of entry are available for citizens to express themselves. The manifestations are digitally registered, containing information about the characteristics of the user’s profile and the manifestations presented.

The first dataset presents the different characteristics of the manifestations carried out by the population, while the second presents the sociodemographic profile of the users that carried out these manifestations.

The data were obtained through a partnership between the General Ombudsman of the Brazilian Unified Health System (Ministry of Health of Brazil), the Regional Office of Aggeu Magalhães Institute, and the Platform of Data Science applied to Health (both from the Oswaldo Cruz Foundation).

Ombudsman’s offices are institutional bodies that aim to make public services responsive to citizens’ demands. Through the ombudsman, the citizen ceases to act as a mere public service user to exercise the role of controller and evaluator of public policies [1].

These data are relevant to public administration and academic research, providing a temporal portrait of the Brazilian population’s aspirations concerning public health.

### Data description

This data paper presents two datasets: the first depicts the characteristics of the manifestations registered by the General Ombudsman, where each row represents an individual manifestation and contains information such as registration date, classification, input channel, and subject, among others; the second dataset is constituted of sociodemographic information for each citizen that performed a manifestation, and characteristics such as the sexual orientation, race, age, and geographic location of the citizen, among others, are presented.

These datasets are generated by acquiring the original microdata and applying specific data construction steps. A detailed description of the methodology can be found in the file etl_methodology. The datasets represent information collected by the Genaral Ombudsman from 2010–01-01 to the day that the microdata is extracted from the source database, which is performed almost on a daily basis. These datasets and related files are shown in Table 1.

**Table 1:** Overview of data files/datasets

Label	Name of data file / dataset	File types (file extension)	Data repository and identifier	References
Data file 1	CITIZEN_PROFILE_20100101_20220719	Compressed Delimited text (.zip)	Synapse: https://doi.org/10.7303/syn31945851	[2]
Data file 2	CITIZEN_PROFILE_20100101_20220719_T	Compressed Delimited text (.zip)	Synapse: https://doi.org/10.7303/syn31945851	[2]
Data file 3	Citizen_profile_updated_dataset	Compressed Delimited text (.zip)	Synapse: https://doi.org/10.7303/syn31945851	[2]
Data file 4	Data_construction_citizen_profile	Jupyter notebook (.ipynb)	Synapse: https://doi.org/10.7303/syn31945851	[2]
Data file 5	Dict_citizen_profile.csv	Delimited text (.csv)	Synapse: https://doi.org/10.7303/syn31945851	[2]
Data file 6	Municipalities.zip	Compressed Delimited text (.zip)	Synapse: https://doi.org/10.7303/syn31945851	[2]
Data file 7	MANIFESTATIONS-20100101_20220719	Compressed Delimited text (.zip)	Synapse: https://doi.org/10.7303/syn31945851	[2]
Data file 8	MANIFESTATIONS-20100101_20220719_T	Compressed Delimited text (.zip)	Synapse: https://doi.org/10.7303/syn31945851	[2]
Data file 9	Manifestations_updated_dataset	Compressed Delimited text (.zip)	Synapse: https://doi.org/10.7303/syn31945851	[2]
Data file 10	Data_construction_manifestations	Jupyter notebook (.ipynb)	Synapse: https://doi.org/10.7303/syn31945851	[2]
Data file 11	Dict_manifestations.csv	Delimited text (.csv)	Synapse: https://doi.org/10.7303/syn31945851	[2]
Data file 12	etl_methodology	Portable document (.pdf)	Synapse: https://doi.org/10.7303/syn31945851	[2]

### Data acquisition

One of the main contributions of this data paper is facilitating access to OUVSUS microdata. Due to security reasons, accessing the DATASUS databases that store this microdata is impossible. Therefore, it was necessary to develop an indirect approach to acquire the data, where an Ombudsman technical staff, with access privileges to the databases, is responsible for manually extracting the desired information. This process is performed on an (almost) daily basis and results in two files: OUVIDORIA_MANIFESTACOES_<extraction_date>.csv and OUVIDORIA_PERFIL_<extraction_date>.csv, which can be obtained in the links manifestation_updated_dataset and citizen_profile_updated_dataset, respectively.

### Data construction

After the data acquisition, the original microdata is cleansed, transformed, and enriched by applying different data construction operations. The definition of the operations is based on detailed studies of the datasets and feedback received from the Ombudsman’s technical staff and specialists in the field.

A general overview of the data construction operations performed for each dataset is presented below:

- *Manifestations* replacement of values in date-typed columns with NULL values when the format is not correct or when the values represent dates outside the coverage period; generation of new columns derived from date columns; fixing incorrect values based on feedback obtained from specialists.
- *Citizen Sociodemographic Profile* renaming of columns due to wrong or archaic titles; fixing values through the application of mapping operations; replacement of invalid age values with NULL; generation of new columns derived from date columns; enrichment with municipality info, such as municipality name, area, coordinates, geographic regions, among others. The information used for the municipality info can be found in the file municipalities.

A detailed description of the data construction process may be found in the notebooks data_construction_citizen_profile and data_construction_manifestations, which exemplify the application to microdata spanning the period from 2010–01-01 to 2022–07-19. The resulting datasets obtained after the data construction process can be found in the files CITIZEN_PROFILE_20100101_20220719_T.zip and MANIFESTATIONS-20,100,101_20220719_T.zip. For reproducibility purposes, the original datasets are found in files CITIZEN_PROFILE_20100101_20220719 and MANIFESTATIONS_20100104_20220719.

A complete description of the variables for each dataset generated after the data construction process can be found in the files dict_citizen_profile.csv and dict_manifestations.csv.

### Updated datasets

The most recent versions of original and derived datasets for manifestations and sociodemographic profiles can be found in the links manifestation_updated_dataset and citizen_profile_updated_dataset, respectively.

## Limitations

- The updated datasets in the links manifestation_updated_dataset and citizen_profile_updated_dataset might contain data extracted from the databases on a previous date to the download process. This occurs due to the indirect extraction approach, where an Ombudsman’s technical staff might not be able to generate the original datasets for a given date.
- There are two reasons for the high number of NULL values in the datasets regarding the characteristics of manifestations. First, some information is acquired only after a registered manifestation has concluded some stages. For example, the variable “DATA DO FECHAMENTO” (conclusion date) is filled only when the corresponding manifestation has gone through all phases necessary to close a register. The second reason for the high number of NULL values is the result of users not providing optional information. This is the case for the variable “BAIRRO DO CIDADAO” (the user’s district that performed the manifestation).
- As the filling of the citizen sociodemographic profile data is the user’s own responsibility, some informations may not have been filled.

## Data Availability

The data described in this Data note (CITIZEN_PROFILE_20100101_20220719, CITIZEN_PROFILE_20100101_20220719_T, citizen_profile_updated_dataset, MANIFESTATIONS-20,100,101_20220719, MANIFESTATIONS-20,100,101_20220719_T and manifestations_updated_dataset) as well as its auxiliary files can be freely and openly accessed on Synapse under the same persistent identifier: https://doi.org/10.7303/syn31945851[2].

## Abbreviations

SUS: Brazilian Unified Health System
DATASUS: Department of informatics of the Unified Health System of Brazil
OUVSUS: General Ombdusman of the Brazilian Unified Health System
